# Implementing effective community-based surveillance in research studies of maternal, newborn and infant outcomes in low resource settings

**DOI:** 10.1186/s12982-021-00109-0

**Published:** 2022-01-12

**Authors:** Caitlin Shannon, Chris Hurt, Seyi Soremekun, Karen Edmond, Sam Newton, Seeba Amenga-Etego, Charlotte Tawiah-Agyemang, Zelee Hill, Alexander Manu, Ben Weobong, Betty Kirkwood, Lisa Hurt

**Affiliations:** 1 Impact and Innovation Team, CARE USA, 115 Broadway, 5th Floor, New York, NY 10006 USA; 2 Centre for Trials Research, College of Biomedical and Life Sciences, Cardiff University, Neuadd Meirionnydd, Heath Park, Cardiff, CF14 4YS UK; 3 Maternal and Child Health Intervention Research Group, Faculty of Epidemiology and Population Health, London School of Hygiene and Tropical Medicine, Keppel Street, London, WC1E 7HT UK; 4 Department of Women and Children’s Health, Kings College London, Strand, London, WC2R 2LS UK; 5 School of Public Health, Kwame Nkrumah University of Science and Technology, Kumasi, Ghana; 6 Kintampo Health Research Centre, Ghana Health Service, Kintampo, Bono East and Bono Regions, Ghana; 7 Institute for Global Health, University College London, 30 Guilford Street, London, WC1N 1EH UK; 8 Department of Epidemiology and Disease Control, School of Public Health, College of Health Sciences, University of Ghana, Legon, P.O. Box LG 25, Accra, Ghana; 9 Department of Social and Behavioural Sciences, College of Health Sciences, University of Ghana, Accra, Ghana; 10 Division of Population Medicine, School of Medicine, Cardiff University, Neuadd Meirionnydd, Heath Park, Cardiff, CF14 4YS UK

**Keywords:** Maternal, Neonatal, Infant, Research, Randomised controlled trials, Population-based, Community, Surveillance

## Abstract

**Background:**

Globally adopted health and development milestones have not only encouraged improvements in the health and wellbeing of women and infants worldwide, but also a better understanding of the epidemiology of key outcomes and the development of effective interventions in these vulnerable groups. Monitoring of maternal and child health outcomes for milestone tracking requires the collection of good quality data over the long term, which can be particularly challenging in poorly-resourced settings. Despite the wealth of general advice on conducting field trials, there is a lack of specific guidance on designing and implementing studies on mothers and infants. Additional considerations are required when establishing surveillance systems to capture real-time information at scale on pregnancies, pregnancy outcomes, and maternal and infant health outcomes.

**Main body:**

Based on two decades of collaborative research experience between the Kintampo Health Research Centre in Ghana and the London School of Hygiene and Tropical Medicine, we propose a checklist of key items to consider when designing and implementing systems for pregnancy surveillance and the identification and classification of maternal and infant outcomes in research studies. These are summarised under four key headings: understanding your population; planning data collection cycles; enhancing routine surveillance with additional data collection methods; and designing data collection and management systems that are adaptable in real-time.

**Conclusion:**

High-quality population-based research studies in low resource communities are essential to ensure continued improvement in health metrics and a reduction in inequalities in maternal and infant outcomes. We hope that the lessons learnt described in this paper will help researchers when planning and implementing their studies.

## Background

Research evidence has contributed to improvements in the health and wellbeing of women and infants worldwide, through an understanding of the epidemiology of key outcomes and the development of effective interventions. It has also identified important knowledge gaps. Maternal and newborn mortality and stillbirths remain unacceptably high in many settings, largely due to preventable causes [1–3] and lack of access to quality healthcare [4]. Monitoring of maternal and child health outcomes for milestone tracking requires the collection of good quality data over the long term, which can be particularly challenging in poorly-resourced settings. Well-designed research studies can help to address these challenges, including prospective observational studies to monitor trends and quantify inequalities, and intervention studies to inform and evaluate new policies and programmes [4, 5].

Establishing pragmatic population-based surveillance systems with high-quality fieldwork, data management, and monitoring is essential to the success of such research studies. Resources exist that provide guidance on the design, and implementation of field studies in low-resource settings (for example, Smith, et al. [6]). These include detailed information on developing a collaborative relationship with the community; identifying and training field staff; enumerating the population; and identifying and mapping eligible households [7, 8]. Advice is also available on the planning and organization of fieldwork, including field structures and supervision; and developing robust data management systems [9, 10]. We have summarised these general issues in Table 1 because they are fundamental to all study designs.

**Table 1:** General considerations when establishing a population-based surveillance system for community-based research studies

• How will the study area be selected?
• How will a collaborative relationship be developed and maintained with the community in the area?
• Is there an up-to-date census of the population and a map of residences?
• How will field staff be selected and trained?
• How will the field staff work together, and what supervisory structures are required?
• Where will field staff be based, and who will manage the logistics of any field offices and any staff accommodation requirements?
• Where will data collection and/or intervention delivery visits take place, and what procedures will be in place for individuals who are not present when a fieldworker calls to collect data?
• What supervisory structures will be in place for field staff, and how will these be monitored and evaluated?
• How will the information be transferred from the field to the central database, and how will the confidentiality of the information be maintained?
• What checks are needed in the field office (before information is sent/uploaded to the central office)?
• Who will develop the data management system, when will this be done, and how will it be tested?
• What data checks and data reports are required on a regular basis, and who will monitor and act on these (to identify areas for adaptation or improvement)?
• Is there capacity for the data management system to evolve to support implementation, and who will be responsible for this?

See [6] for more detail on each aspect

Despite this wealth of general advice, there is a lack of specific guidance on designing and implementing studies of mothers and infants. Additional considerations are required when establishing surveillance systems to capture real-time information at scale on pregnancies, pregnancy outcomes, maternal and infant health outcomes for research. This paper harnesses two decades of research collaborations between Kintampo Health Research Centre in Ghana (KHRC) and the London School of Hygiene and Tropical Medicine (LSHTM) to provide structured guidance to researchers planning community-based studies in low-resource settings. Based on the experience of the teams who designed and ran the ObaapaVitA, Newhints and Neovita trials [11–13], we propose a checklist of key items to consider when setting-up and implementing research studies that require pregnancy surveillance and identification and classification of maternal and infant outcomes.

## Summary of ObaapaVitA, Newhints and Neovita

Table 2 summarises the design of the surveillance systems and fieldwork procedures in these trials. Briefly, ObaapaVitA was a cluster-randomised, placebo-controlled trial to examine the effect of weekly low-dose vitamin A supplementation given to women of reproductive age on maternal and infant outcomes. Newhints was a cluster-randomised controlled trial to examine the effect of a home-visiting strategy on neonatal mortality and newborn care practices. Neovita was an individually-randomised, placebo-controlled trial to examine the effect of newborn vitamin A supplementation on post-supplementation mortality and infant hospitalisations.

**Table 2:** Description of the surveillance systems used in the three trials

Trial name	ObaapaVitA	Newhints	Neovita
Trial design	Cluster-randomized, double-blind, placebo-controlled	Cluster-randomized, usual care controls	Individually-randomized, double-blind, placebo-controlled
Eligible population	All women in women aged 15–45 years living in seven rural districts in Brong Ahafo Region in Ghana capable of giving informed consent and planning to live in the trial area for at least 3 months		Neonates who were at least two hours old; on the day of birth or in the next two days; able to feed orally; and likely to stay in the area for at least 6 months
Dates of data collection	Phased start by site: 12/2000–10/2008 (Kintampo N and S) 06/2001–10/2008 (Wenchi and Tain) 06/2002–10/2008 (Techiman) 01/2003–10/2008 (Nkoranza N & S)	Surveillance system that had been established for ObaapaVitA continued from 11/2008 to 12/2009	Enrolment started in 08/2010 Last infant followed to 12 months of age at 11/2012
Frequency of visits	Every 4 weeks, to all enrolled women	Every 4 weeks to all enrolled women In July 2009, because of budget constraints, this frequency was reduced to visits every 8 weeks and restricted to women who were pregnant and infants	All reproductive age women visited every 12 weeks All pregnant women visited every 4 weeks until 8th month of pregnancy and daily in the last month of pregnancy All infants visited every 4 weeks until status of infant at 12 months of age was ascertained
Data collection team	Fieldworkers, who were supervised by site leader, co-ordinators, and supervisors; senior supervisors for more complex data collection; dosing supervisors in Neovita		
Tasks completed during fieldworker visits	1. Collect 4-weekly data 2. Distribute intervention and control capsules 3. Enrol new eligible women (e.g., if they had moved in or turned 15)	1. Collect 4 weekly data 2. Enrol new eligible women (e.g., if they had moved in or turned 15)	1. Collect 4- or 12-weekly data
Primary outcome(s)	1. Pregnancy-related mortality 2. All-cause mortality in women of reproductive age	1.All-cause neonatal mortality	1. Post-supplementation infant mortality to 6 months of age
Secondary outcomes	1. Severe maternal morbidity 2. Stillbirths 3. Perinatal mortality 4. Neonatal mortality 5. Infant mortality	1. Age-specific neonatal mortality 2. Cause-specific neonatal mortality 3. Behavioural outcomes (that is, how many women practiced the Newhints behaviours)	1. Post-supplementation neonatal mortality 2. Post-supplementation infant mortality 3. Post-supplementation neonatal morbidity 4. Adverse events
Other data collection or visits	1. Additional forms completed by supervisors with additional training (e.g., verbal autopsies, adherence) 2. Hospital based surveillance (from 01/2004), to collect data on maternal morbidities	1. Verbal autopsy forms completed by supervisors with additional training 2. In intervention zones, CBSVs aimed to visit women twice during pregnancy and three times after birth (on days 1, 3, and 7)	1. Verbal autopsy forms completed by supervisors with additional training 2. Dosing supervisors gave the study capsules to enrolled newborns in the community and in birthing facilities, and collected adverse event data on days 1 and 3

These were complex trials. They provide valuable insights for future research because they:

1. Had very large sample sizes (field staff in ObaapaVitA visited 120,000 women every month, making more than 8 million home visits in almost eight years of fieldwork);
2. Examined rare outcomes (the primary analysis for ObaapaVitA was based on 286 pregnancy-related deaths in almost 80,000 pregnancies);
3. Integrated the wider health system into trial implementation (the Newhints home visits intervention was designed jointly with 7 district health management teams (DHMTs) and delivered by existing Community-Based Surveillance Volunteers [CBSVs] with monitoring data collected by supervisors based within the DHMTs); and
4. Required genuine real-time data (the 22,955 infants in the Neovita had to receive the neonatal supplement within 72 h of birth).

Table 3 summarises the key lessons learnt from these studies. These are grouped under four headings, with an accompanying checklist of items to consider and possible solutions. Whilst many of the issues are relevant to studies in any field, our focus is on circumstances where complete data on pregnancies and their outcomes is a requirement.

**Table 3:** Lessons learnt and accompanying checklist for planning the design and implementation of community-based maternal, newborn and infant health studies

	Checklist of items to consider at the planning stage:	Solutions
1. Understand your population	*What are the cultural and social norms on disclosing a pregnancy in the population?* *What are the cultural and social norms and behaviours relating to delivery in the population?*	• Conduct formative research to understand the norms around disclosing a pregnancy in your population • Consider fieldworker characteristics, location, training, and supervision, and how these might affect a woman’s likelihood to discuss their pregnancy with them • Conduct formative research to understand behaviours around delivery (in particular, whether women move shortly before and after delivery and how they make plans for delivery) • Collect data from women on their delivery plans (including planned place of residence and place of delivery), so that data collection and intervention delivery issues can be mitigated
2. Plan your data collection cycle	*How frequent should study visits be?* *Does the frequency of visits need to change as participants progress through the study?* *Do you need other methods of data collection to improve detection rates in between visits?* *Do you need to recruit the mothers before they give birth?* *When will study visits end?*	• Plan for study visits that are as frequent as resources allow • Consider intervention delivery requirements in addition to data collection requirements • Consider the issues that might lead to changes in visit frequency as participants progress through the study (e.g., instigate more frequent visits once a woman is pregnant, or in the later stages of a pregnancy if births need to be detected quickly) • Consider alternative methods of data collection (e.g., contact with key informants, mobile phone use) • Consider whether women need to be enrolled in the surveillance system before the pregnancy outcome (e.g., this may be essential for intervention delivery in some cases, but not in others) • Plan the last visit at a reasonable time after the end of the period of interest (e.g., to capture maternal outcomes at the end of the 6-week postpartum period, plan the visit for the 8–12th week or later if the data collection is not time-sensitive)
3. Enhance routine surveillance with additional data collection methods	*Can you use data from multiple sources to identify pregnancies and outcomes, and for triangulation?* *Have you developed a strategy for dealing with inconsistencies between different sources?* *Is additional confirmation needed for some data?*	• Consider which additional data sources are useful, including: -Data collection at hospitals or clinics (e.g., for data on morbidities; also good for identifying pregnancies not reported in the field and for confirming dates) -Verbal autopsies (for cause of death information; also good for identifying pregnancies not reported in the field and for confirming dates) • Include resources to employ, train and supervise senior staff to collect data form these additional sources • Incorporate strategies for dealing with inconsistencies between sources a priori into the data cleaning plan • Consider whether additional confirmation is needed for some data (e.g., is a woman’s self-report of a pregnancy adequate or is formal pregnancy testing needed?)
4. Design a field and data management system that is adaptable in real-time	*Which events should trigger changes in data collection or intervention procedures?* *Have you developed a mechanism for reporting and dealing with data errors, in real-time if necessary?*	• Consider whether data collection will be different at different points in the study, and how this can be changed in real-time (e.g., do you want to collect more data from women once they are pregnant?) • Consider whether there needs to be regular changes to work listings (e.g., do you want to collect data on mothers and infants after birth, and should the infant appear on a fieldworker’s work listings?) • Allow for appropriate intervals in the production of work listings (e.g., allowing for mourning periods before collecting verbal autopsy data) • Consider the specific errors that may occur with data collection on mothers and infants and develop a plan that will allow for the correction of these in real-time

## Understand your population

To plan an appropriate surveillance system and intervention delivery mechanisms, it was essential to understand the cultural and social norms and behaviours relating to pregnancy, delivery, and infant care practices in the population under study. Extensive formative research was conducted to establish the ObaapaVitA trial surveillance system (which formed the basis for the subsequent Newhints and Neovita trials) before fieldwork began, to explore these issues as well as factors that may have affected adoption or adherence to the interventions of interest [14–16].

### What are the cultural and social norms on disclosing a pregnancy in the population?

Establishing when a woman is pregnant, especially in the early stages, is difficult [17]. In common with other settings, women in this study area were reticent to discuss early pregnancy losses and induced abortions [15]. Although these norms posed challenges (for example, women were on average between five and 6 months pregnant when they revealed their pregnancies to the fieldworkers), the most effective mitigation strategies related to the characteristics, location, supervision, and training of fieldworkers. Wherever possible, fieldworkers visited the same women for the duration of fieldwork, to develop trust. They lived in the areas in which they worked, becoming integrated into the community and learning about outcomes that occurred between routine surveillance visits. Ethnicity and religion were considered when posting fieldworkers into communities, also acknowledged as important by others [7]. Whether fieldworkers were women or men was not influential in this setting, but may be important to consider in other settings [18].

The need to develop good relationships with the women and their families was emphasised during fieldworker training. Sessions on communication skills and ethical behaviour within research were included [19]. Fieldworkers were trained to interview women in locations where they could not be overheard wherever possible. Ongoing training was provided through weekly field meetings and routine field supervision, and refresher sessions were provided at least once per year in response to feedback received from communities and through continuous data quality reviews. For example, we developed training sessions that aimed to increase the completeness and sensitivity of ascertainment of information on early pregnancy losses and pregnancy-related deaths using novel methods, such as discussions on the impact of maternal deaths on a family and role playing.

### What are the cultural and social norms and behaviours relating to delivery in the population?

Behaviours and practices around birth and the postpartum period also affected timely data collection and intervention delivery. Whilst the influence of population mobility on global disease epidemiology [20] and on loss to follow-up in surveillance systems [21] are well-understood, the issues caused by temporary migration within studies is less widely discussed. In this study area, migration in the later stages of pregnancy was common, as women moved to be closer to a healthcare facility or to stay with relatives for the birth. Fieldwork practices had to be adjusted so that information on the woman’s study identification number was collected and verified at each visit. This ensured that data from their new location could be linked to their previous surveillance data, and the outcome was not counted again when the woman moved back to her original household.

In ObaapaVitA, this migration had important implications for intervention delivery of either vitamin A or placebo capsules. It was not possible to ensure that women received supplements from the same trial arm as in their previous residence because of the cluster randomisation, the low population density, and large area involved. This resulted in a change in treatment group in approximately half of women who moved, and reduced the numbers eligible for inclusion in the primary analysis (see the results paper for a detailed discussion of these issues [11]). In contrast, this was not an issue for Neovita, as this was an individually randomised controlled trial where the intervention was a single dose shortly after birth.

Exploring migration practices around delivery in a study area during the formative stage of a study and collecting data on women’s delivery plans (including planned place of residence at that time and intended place of delivery) can mitigate these issues, an approach implemented in Newhints which allowed for changes to be made to intervention implementation.

## Plan your data collection cycle

To capture information on all pregnancies in the study population, we recruited all women of reproductive age into a regular surveillance system. This allowed for complete and timely identification of pregnancies; accurate date measurements (for gestational age and time-based definitions); rapid identification of births (in Newhints and Neovita); and complete and timely intervention delivery including in the antenatal period (in ObaapaVitA and Newhints). Fieldwork procedures to ensure this, including issues such as visit frequency and additional data collection mechanisms, are described below.

### How frequent should study visits be?

Determining the frequency of study visits involved resolving the tension between an ideal schedule and available resources. It has been suggested elsewhere that visits are required at least every 3–6 monthly to capture “*reasonable*” information on births and deaths [8]. However, in ObaapaVitA and Newhints, fieldworkers visited all women in the surveillance system every 4 weeks. The rolling programme of visits allowed data to be collected, processed and work “listings” for the next visit to be produced prior to that visit (Table 4). Although such regular visits had major resource implications—these projects employed more than 360 field staff at any one time—they resulted in more pregnancies and pregnancy-related deaths captured in the surveillance than those captured by the surveillance systems of the local District Health Management Teams (which relied on six monthly sweeps) (data not published, documented in minutes of the ObaapaVitA Trial Steering Committee meeting 2006). This was partly because they ensured relationship-building between fieldworker and participant. In addition, we could ask women regularly (and in a way that aligned with a typical menstrual cycle) about pregnancies, which enabled improved recall accuracy as they only had to remember events that had occurred over the last month.

**Table 4:** Data collection cycle

Activity	Week 1	Week 2	Week 3	Week 4
Data collection	FW area 1	FW area 2	FW area 3	FW area 4
Data checks in field office	FW area 4	FW area 1	FW area 2	FW area 3
Data entry; verification; R and C checks	FW area 3	FW area 4	FW area 1	FW area 2
IDBCs; listings and labels printed	FW area 2	FW area 3	FW area 4	FW area 1

*FW* fieldworker (each fieldworker was responsible for 4 fieldwork areas), *R and C* range and consistency, *IDBCs* interdatabase checks (for example, checking consistent dates of birth in different databases)

Intervention delivery requirements also influenced fieldwork set-up and visit frequency. In ObaapaVitA, the intervention consisted of capsules that were taken once per week. However, visiting more than 200,000 women weekly to distribute and observe capsule taking was not feasible. Combining capsule delivery with 4-weekly data collection was a feasible alternative and meant that women were given a manageable number of capsules (four) at each visit and that adherence could be checked regularly, both by self-report and by checking the number of remaining capsules. Formative work also found that women preferred it if all participants took their capsules on the same day of the week, as this could be accompanied by clear and simple messaging and meant that participants could remind each other [14].

### Does the frequency of visits need to change as participants progress through the study?

In Neovita, the study intervention (vitamin A or placebo) was given to neonates within 72 h of birth. Therefore, it was possible to have longer intervals between fieldwork visits of non-pregnant women, with home visits every 3 months at which pregnancy status was ascertained. However, fieldwork visits needed to increase towards the end of a pregnancy, to ensure that births were identified quickly. All pregnant women were therefore visited every 4 weeks until the eighth month of pregnancy, and then daily in the last month of pregnancy to identify births. Other strategies were used to complement the field activities, including daily sweeps of villages (especially where women were thought to be close to delivery) and health facilities by supervisors, and recruitment of key community informants to report births to the field team as soon as they happened. In combination, these ensured that three-quarters of the 22,955 neonates recruited received the intervention within 24 h of birth, and 99.8% within 72 h of birth.

Visits were also required on days one and three post-dosing to assess for adverse events. This was challenging, because women could be discharged home from hospital during this period. The work could not be planned using electronic listings because the field offices did not have adequate computing equipment or access to the internet. Instead, a system of paper-based “dosed infants” listings were prepared by the dosing team every night once the numbers and locations of dosed infants were known. This intensive system ensured that data on adverse events within three days of supplementation were collected for 99.5% of neonates in the trial [13].

Early postpartum visits were key to the delivery of the Newhints intervention. These visits were conducted by more than 400 CBSVs in 49 intervention zones employed by DHMTs and managed independently to the surveillance data collection of outcome measures conducted by KHRC field workers. The trial was designed to estimate the effects of the intervention under real-world conditions; although pregnancies and births were identified within the 4-weekly surveillance system, this information was not shared with the CBSVs. They were responsible for this identification as they would be in a routine programme. The coverage of intervention visits achieved by the CBSVs was lower than the coverage intervention delivery achieved by ObaapaVitA or Neovita; 63% of women reported having at least one postpartum visit from a CBSV. Researchers therefore need to be clear whether their work is aiming to understand the efficacy (as in Neovita) or effectiveness (as in Newhints) of an intervention when planning the methods of delivery and intensity of implementation and fieldwork required. Note that it is always important to have outcome data collected by fieldworkers either ignorant to trial arm as in a placebo-controlled trial such as ObaapaVitA or Neovita, or independent of those delivering the intervention such as in Newhints.

### Do you need other methods of data collection to improve detection rates in between visits?

Where there are insufficient resources for frequent surveillance, researchers will need to consider augmenting data collection using other methods, which can include mobile phone interviews, key community informants or outcome identification in health facilities. All of these were used in Neovita, contributing to very high (98.9%) data completeness for the primary outcome (infant mortality at six-months’ age). These methods work well for outcomes such as births or deaths, but are less useful for outcomes prone to recall or other biases such as data on healthcare utilization, or for collecting sensitive information such as data on pregnancy loss.

### Do you need to recruit the mothers before they give birth?

Intervention delivery requirements also determine whether women need to be enrolled before the pregnancy outcome, or whether they and their infants can be recruited after birth. For example, recruitment at birth worked in Neovita. In fact, combining both pregnancy surveillance and ‘active’ birth ascertainment was essential to reduce the selection bias that would be introduced by only recruiting infants born in facilities or the infants who were easiest to reach quickly after birth. For all three trials, we also know that fewer infant outcomes would have been captured using a post-birth only surveillance model because of the migration of women for delivery described above. Community surveillance of, and relationship-building with, pregnant women also had important implications for ascertainment of perinatal outcomes (for example, allow careful interviewing about whether the baby that cried moved or breathed for a short time after birth to distinguish between stillbirths and early neonatal deaths), permitting accurate ascertainment of both numerator and denominator information. Lastly, some interventions may require preconception visits, which can only be achieved if all women of reproductive age are recruited into surveillance and may require additional questions within the surveillance about pregnancy intentions.

### When will study visits end?

Maternal and infant outcomes have time-based definitions [5]. There must be at least one planned visit after the end of these time periods to minimise loss to follow-up. This is easier with ongoing surveillance, but we also continued some data collection activities after surveillance ended to ensure satisfactory data completeness. For example, follow-up of infants in Newhints was completed in December 2009, but verbal autopsy (VA) data collection continued for a further 12 months with a reduced workforce. This also happened in Neovita, along with multiple targeted visits to obtain infant status information. Staffing plans need to include provision for these essential ‘mop-up’ activities, with consideration given to staff numbers and grades. In both Newhints and Neovita, these final follow-up visits became the responsibility of supervisors who had motorbikes (and could travel longer distances) and were familiar with the study area.

## Enhance routine surveillance with additional data collection methods

Regular household visits by fieldworkers may not be enough to ensure complete and/or accurate information on pregnancies and their outcomes. We used several additional data sources to augment the data collection. Senior supervisors were stationed on the maternity wards of the four local district general hospitals in ObaapaVitA and Neovita. There was no civil registration and vital statistics system in the study area at the time for data linkage. Therefore, VAs were conducted in each study to obtain cause of death information.

### Can you use data from multiple sources to identify pregnancies and outcomes, and for triangulation?

Both the hospital data collection and VAs allowed for the identification of pregnancies and deaths not reported elsewhere, especially early pregnancies (of particular importance in ObaapaVitA). Completeness of birth capture was significantly enhanced by supervisors in Neovita visiting health facilities daily.

Combining information from different sources was also important for triangulation. Dates of birth and deaths could be checked (for example, to confirm whether deaths fulfilled the time-based definitions as required for outcome classification), as well as other important information for outcome definition (for example, whether the baby cried or moved at birth to distinguish between stillbirths and early neonatal deaths). Information on maternal and neonatal morbidity (which are known to be poorly recalled and recognized by women, [22, 23]) were also confirmed using these sources.

In addition, the diagnostic accuracy of a VA tool for ascertaining causes of stillbirths and neonatal deaths was assessed by comparing the coded information from the VAs with hospital cause of deaths information for a year’s worth of data in ObaapaVitA trial [24]. Overall, the diagnostic accuracy of the VAs was higher than expected for neonatal deaths, with sensitivity > 60% for all major causes and specificity of 76% for birth asphyxia and > 85% for prematurity and infection. The VA performed poorly for stillbirth diagnoses such as congenital abnormalities and maternal haemorrhage.

Triangulation of data from different sources therefore allowed for improved data quality, but was also challenging, in terms of the resources needed to recruit more senior staff, provide additional training, ensure close and regular supervision of field staff, and data management requirements.

### Have you developed a strategy for dealing with inconsistencies between different sources?

We pre-specified data cleaning plans for investigating and resolving inconsistencies between the different data sources. In ObaapaVitA, the aim was to maximise the detection of pregnancies and pregnancy-related deaths in recruited women. Our plan therefore allowed for the inclusion of pregnancies identified by the additional data collection sources but equally, if we knew from the community surveillance that a woman was pregnant or had recently delivered when she died, this information was given more weight than a VA report that had failed to identify the pregnancy.

Care and sensitivity were required when incorporating these data into study databases. For example, we often knew from the hospital data that a study woman had been admitted early in pregnancy, before the pregnancy had been reported to their fieldworker. It was important to capture these pregnancies and any associated outcomes. However, the hospital data was not used to update the study databases from which the fieldwork listings was generated, because the pregnancy may have ended during the admission or because the woman may not have told other household members about the pregnancy.

### Is there a need for objective confirmation of data?

Some studies require formal confirmation of data reported by women. For example, intervention delivery might not start until the woman has a positive pregnancy test (as has been done in some trials of antenatal micronutrient supplements, for example, [25, 26]). Outcomes may also need additional confirmation, such as the need for blood pressure and urine protein measurements for the confirmation of hypertensive diseases in pregnancy [27]. If required, these methods are resources-intensive and careful planning is required to determine how samples can be taken and accurate measurements can be ensured.

## Design a field and data management system that is adaptable in real-time

Data collection and management systems for clinical trials need to be capable of processing large amounts of data efficiently. They also need to be adaptable, both to changes in the status of study participants and changes in study protocols [28]. In our trials, changes to participant status in the databases (such as pregnancy, delivery or migration) were integrated within the four-week data collection and processing cycle as described below.

### Which events should trigger changes in data collection or intervention procedures?

In the surveillance system, most of the women were not pregnant for the majority of the surveillance period. However, when a woman reported a pregnancy, we needed to ask them different questions (as happened in ObaapaVitA), change how often they were visited (as happened in Neovita), or introduce new elements to the fieldwork (for example, delivering the intervention in Neovita). All three trials used work listings that were updated automatically every week after data entry, with any necessary changes activated before the next visit was due (Table 4). This ensured that surveillance fieldworkers had the correct forms at the next visit for women who had disclosed a pregnancy or whose pregnancies had ended, and this information was incorporated into the work schedule of the appropriate staff member. We also included additional rules within the production of these listings, including that no request for a VA appeared before the end of the standard 6-week/40-day mourning period.

### Have you developed a mechanism for reporting and dealing with data errors, in real-time if necessary?

Although field errors were kept to a minimum through close supervision and intensive field checks, extensive data cleaning efforts were required due to the overall volume of field visits. Flexible and automated mechanisms to ensure continuous feedback from the field to the data processing centre, and vice versa, were essential to ensure that errors and inconsistencies were corrected in real-time if possible. For example, errors were occasionally made in recording infant identification numbers which resulted in the baby being linked to the wrong mother in the database and therefore a fieldworker being asked to complete the wrong forms. Fieldworkers submitted errors and solutions required weekly to the data processing centre via formal “problem forms”, allowing for immediate correction. This was important to ensure that encounters that may be distressing to study participants were prevented. For example, two women in the same household may have given birth around the same time. If one child died but identification numbers of the babies were mixed-up, it was essential that this was resolved quickly so that the grieving mother was not repeatedly asked for details about her baby. A similar situation could arise with twins or triplets where only one infant survived the pregnancy. Fieldworkers were trained to deal with these issues with sensitivity, but a well-functioning data management system was also essential to ensure that these problems did not re-occur.

We also encountered other unexpected errors, including pregnancies lasting 10 months or more in duration and two pregnancies in the same woman that were too close together. Checks were therefore developed whilst data collection was ongoing to flag these issues. The best solution for these was that they were investigated and resolved by senior field staff, but strategies were also developed to check and clean any remaining errors at the analysis stage. For example, we identified several instances of women reporting an early pregnancy loss and then would give birth to a healthy live born infant of a reasonable birth weight 6 months or so later (suggesting that they were not significantly premature). In these instances, we assumed that there was no early pregnancy loss (therefore removing this outcome from the denominator). It was harder to resolve the issues with the pregnancies that were excessively long. In some instances, women delivered healthy infants at the end of these pregnancies. We assume that some therefore experienced early pregnancy losses followed quickly by another pregnancy, but it was rare that we could corroborate this (even within these intensive surveillance systems). Nevertheless, it was important to understand these potential errors so that any inconsistencies between the study data and other data sources could be interpreted and contextualised.

## Discussion

In this paper, we have summarised advice for researchers designing and implementing field studies of maternal and infant outcomes under four key headings: understanding your population; planning data collection cycles; enhancing routine surveillance with additional data collection methods; and designing data collection and management systems that are adaptable in real-time.

There are some limitations to the generalisability of the advice. These were all intervention studies, and many of the lessons learnt arose from understanding the inter-relationship between field procedures for data collection and intervention delivery. However, many of the principles discussed are useful for planning observational studies which also require high-quality data collection and management via complex and complete community-based surveillance systems. Data collection was paper-based, with relational databases updated weekly to ensure that the mothers and infants were visited at the right time and appropriate actions were taken during the visits. Electronic data collection is now increasingly common. However, there are still valuable lessons to be learnt from reflecting on the field set-up in these trials because—whilst some of the issues are resolved by electronic data capture—others are not or may be exaggerated. For example, with paper forms, it was straightforward to compare reported births with registers of pregnancies that had been collated in the field office and therefore investigate and correct errors (especially in the identification number on the new birth form, which had to be completed by hand). These errors may not be picked up until the next set of visits when data are inputted directly into an electronic data capture system.

Both the policy and research environments have changed since these studies were conducted. Many important research questions in maternal and child health remain unanswered but obtaining funding for such intensive studies (which are expensive to set-up and manage) remains challenging. Routinely-collected data is becoming more available in low-resource settings, including improved vital registration systems [29], health management information systems within facilities, and community health information systems that organise information on individuals and families [4]. There are also more established demographic surveillance systems [30] and household survey programmes [31] within which studies can be embedded. Examples of studies that have successfully linked demographic and clinical surveillance systems are increasing [32]. These data sources increase the feasibility of large research studies by providing contextual information, data on population parameters required for study design, and an existing infrastructure which may reduce study costs. Collaboration is mutually beneficial because investment and support for capacity development from research can also help to evaluate and improve the quality and sustainability of these routine monitoring systems.

High-quality population-based research studies in low resource communities are essential to ensure continued improvement and a reduction in inequalities in maternal and infant outcomes. We hope that the lessons learnt described in this paper helps investigators to build and develop on our experiences when planning and implementing their studies.

## Data Availability

Not applicable.

## Abbreviations

CBSV: Community-based surveillance volunteers
DHMT: District Health Management Team
KHRC: Kintampo Health Research Centre
LSHTM: London School of Hygiene and Tropical Medicine
VA: Verbal autopsy
